# Aerobic Exercise and Human Visual Cortex Neuroplasticity: A Narrative Review

**DOI:** 10.1155/2022/6771999

**Published:** 2022-07-23

**Authors:** Dania Abuleil, Benjamin Thompson, Kristine Dalton

**Affiliations:** ^1^School of Optometry and Vision Science, University of Waterloo, Waterloo, ON, Canada; ^2^Center for Eye and Vision Research, Hong Kong, Hong Kong

## Abstract

There is compelling evidence from animal models that physical exercise can enhance visual cortex neuroplasticity. In this narrative review, we explored whether exercise has the same effect in humans. We found that while some studies report evidence consistent with exercise-induced enhancement of human visual cortex neuroplasticity, others report no effect or even reduced neuroplasticity following exercise. Differences in study methodology may partially explain these varying results. Because the prospect of exercise increasing human visual cortex neuroplasticity has important implications for vision rehabilitation, additional research is required to resolve this discrepancy in the literature.

## 1. Introduction

The visual cortex exhibits pronounced neuroplasticity during a “critical” or “sensitive” period of early development [1]. After the closure of this critical period, which in humans is estimated to occur at approximately age 7 [2], visual cortex neuroplasticity is attenuated [3]. However, recent evidence in both animal models and humans suggests that adult visual cortex neuroplasticity can be boosted by a range of interventions including environmental enrichment [4, 5], pharmacological manipulation [6–10], noninvasive brain stimulation (see [11] for a comprehensive review, [12–15]), and aerobic exercise [16–18].

Of these various neuroplasticity enhancers, aerobic exercise is perhaps the most attractive because of its general health benefits. There is strong evidence that physical exercise can enhance neuroplasticity in cortical networks ([19]; see [20] for a recent review; [21]). In particular, enhanced cognitive and motor performance has been positively associated with exercise and physical activity [22, 23]. For instance, rodents with access to a running wheel performed better on learning and memory tasks compared to rodents without a running wheel in their cage [24]. Additionally, exercise can protect against the negative physiological and behavioral impacts of stress [25, 26] that include reduced neuroplasticity and neurogenesis ([27]; see [28] for an overview). One potential mechanism for this effect is an exercise-induced increase in production of brain-derived neurotrophic factor (BDNF), a protein that supports and maintains neuronal growth and maturation [29] and enhances neuroplasticity (see [30] for a comprehensive review). However, as described below, while there is also strong evidence that aerobic exercise enhances visual cortex neuroplasticity in animal models, studies involving human participants have produced widely varying results. Here, we review both animal and human studies investigating the effects of exercise on visual cortex plasticity, either alone or in combination with another neuroplasticity modulating technique. We also consider factors that may explain differing results across studies. In particular, we suggest that inconsistencies in the results of human studies may be attributed, in part, to variations in the intensity and amount of exercise, the timing of exercise with respect to the outcome measure, and the psychophysical task used to index neuroplasticity. We conclude by suggesting ways to advance the field.

## 2. Measures of Visual Cortex Neuroplasticity in Animal Models

Common measures of visual cortex neuroplasticity in animal models involve deprivation of one eye [31]. When visual cortex neuroplasticity is high, monocular deprivation causes a pronounced shift in visual cortex ocular dominance whereby the proportion of cells that show a larger response to stimulation of the nondeprived eye relative to the deprived eye increases [1]. The magnitude of this ocular dominance plasticity is typically measured electrophysiologically and can be used as an index of visual cortex neuroplasticity. In addition, monocular deprivation during the critical period causes deprivation amblyopia, a neurodevelopmental vision disorder characterized by poor amblyopic eye visual acuity and loss of binocular vision in animal models [32–34]. Recovery of vision in post-critical-period animals with deprivation amblyopia requires significant visual cortex neuroplasticity. Therefore, the extent of vision recovery measured electrophysiologically or behaviorally can be used to index visual cortex neuroplasticity.

## 3. Effects of Aerobic Exercise on Visual Cortex Neuroplasticity in Animal Models

### 3.1. Physiological Changes

Studies of rodents have revealed several exercise-induced neurochemical and physiological changes within the visual cortex that may enhance neuroplasticity [35–38]. These changes include a general reduction in cortical inhibition within the primary visual cortex [36] as well as an upregulation and activation of neural growth factors such as BDNF, IGF-1, and VEGF [37], all thought to stimulate neurogenesis or neural growth [35]. Physical activity in rodents also enhances pyramidal cell firing in the primary visual cortex which alters the response of cells in V1 to visual input [38, 39] and improves visual cortex response gain [40–42].

### 3.2. Behavioral and Electrophysiological Effects

In addition to the physiological changes that occur within the visual cortex during and after physical exercise, animal studies have also demonstrated effects on visual perception such as improved object recognition [43] and enhanced discrimination of bidirectional moving gratings [44]. There is strong evidence that exercise, particularly voluntary exercise, enhances ocular dominance plasticity in adult rodents measured using in vivo electrophysiology [45–47]. Adult mice in environments with running wheels showed more pronounced ocular dominance plasticity following 7 days of monocular deprivation compared to mice without a running wheel. Furthermore, physical exercise alongside environmental enrichment allowed for the recovery of amblyopia in adult rats [45]. Rats were rendered amblyopic by performing an eyelid suture at P21 followed by a reverse-suture (opening the closed eye and closing the open eye) at P70. Adult rats were then placed in different environments to assess the factors that enhance vision recovery including visual enrichment, motor enrichment, and classic bright conditions, as well as dark conditions. Rats in the exercise condition recovered normal visual acuity and ocular dominance, indicating enhanced neuroplasticity.

## 4. Measures of Visual Cortex Neuroplasticity in Humans

Many studies investigating visual cortex neuroplasticity enhancement in humans utilize visual perceptual learning paradigms, where repeated exposure to a visual task leads to improved task performance [48–51]. Visual perceptual learning is likely to involve training-induced changes at multiple stages of the cortical visual processing hierarchy [52, 53], and interventions that enhance visual cortex neuroplasticity are expected to increase the rate, magnitude, and/or generalizability of learning. Another common index of human visual cortex neuroplasticity involves the use of short-term monocular occlusion to transiently alter ocular dominance. Specifically, patching one eye for two hours has been found to strengthen the contribution of the deprived eye during binocular viewing, with Lunghi et al. [54] being the first group to demonstrate this effect in young heathy adults. Other studies replicated these findings using psychophysical [55, 56] and electrophysiological [57, 58], as well as imaging [59–61] techniques. The magnitude of the ocular dominance shift or, to borrow a term from the animal literature, ocular dominance plasticity can be used as a measure of visual cortex neuroplasticity.

## 5. Effect of Aerobic Exercise in Humans

The ability of exercise to enhance visual cortex neuroplasticity is evident in animal models; however, these effects have yet to be unequivocally demonstrated in human adults, while there is some evidence to support the notion that exercise can augment visual cortex neuroplasticity in healthy young adults [17, 18, 21, 62, 63], other studies report no effect of exercise [16, 64–67] and possibly a counterproductive effect depending on the timing of exercise [68] (see Table 1). Nonetheless, structural and neurochemical changes within the human brain have been associated with exercise [69, 70]. The balance between excitation and inhibition within the visual cortex is thought to modulate neuroplasticity, whereby an increase in excitation promotes plasticity [59, 61, 71, 72]. One way to assess changes in inhibitory tone within the human visual cortex is to measure the relative local concentrations of the inhibitory neurotransmitter gamma aminobutyric acid (GABA) and the excitatory neurotransmitter glutamate using magnetic resonance spectroscopy. Maddock et al. [18] found that exercise increased the concentrations of both GABA and glutamate within the visual cortex suggesting a greater availability of neurochemicals without a change in the balance of excitation and inhibition. Another approach is to measure the amplitude of visually evoked potentials (VEPs) to assess changes in visual cortex excitability. Using this approach, Bullock et al. [73] observed that low intensity physical exercise increased visual cortex excitability (exercise increased VEP amplitude). The extent to which these neurochemical and excitability changes induced by exercise alter visual cortex neuroplasticity remains unclear.

In support of the theory that exercise enhances human visual cortex neuroplasticity, Lunghi and Sale [17] observed that young adults exhibited greater ocular dominance plasticity following exercise and monocular deprivation as compared to rest and monocular deprivation [17]. This effect was demonstrated with a binocular rivalry visual task that involved the dichoptic presentation of orthogonal gratings while participants reported their percept (the left eye grating, right eye grating, or a mixture of both) over the span of 2 hours. The deprived eye dominated perception for a longer period in the exercise group indicating a larger ocular dominance shift presumably enabled by increased visual cortex neuroplasticity.

Recently, Virathone et al. [21] observed that mild-to-moderate cycling completed in 10-minute intervals distributed across 2 hours reduced binocular rivalry eye dominance in young healthy adults. This finding demonstrates that exercise alone without monocular deprivation, visual learning paradigms, or other interventions may impact binocular rivalry outcomes, perhaps by reducing cortical inhibition.

Other studies have investigated the effects of physical exercise on vision and visual cortex neuroplasticity in older adults. An early study found that aerobic exercise enhanced visual attention in older adults aged 65 to 74 as compared to their younger counterparts aged 20-35 [74]. Additionally, several studies have found that although brain volume decreases with age [75], older adults who are more physically active have higher brain volume [76–78]. As cortical function declines with maturation, it appears that physical exercise may counteract or slow the effects of aging. In addition to the older adult population, one study has reported improved vision following physical exercise in patients with amblyopia [79]. Visual acuity and stereoacuity improved after six daily cycling sessions in adults with anisometropic amblyopia [79]. However, these effects may have been due to a parallel intervention (reverse patching) that the participants received during exercise that was not controlled for in the experimental design.

Zhou et al. [16] attempted to replicate the exercise-induced enhancement of ocular dominance plasticity reported by Lunghi and Sale [17] using a binocular combination task. Participants were presented with dichoptic gratings that had the same spatial frequency but different phases and reported the perceived phase when the gratings were fused. The perceived phase is biased towards the phase presented to the dominant eye. The authors found no significant effect of physical exercise on ocular dominance plasticity [16]. In a subsequent study, the group was also unable to replicate Lunghi and Sale's [17] results despite using an identical visual task [21, 67]. An absence of exercise effects has also been reported in studies using EEG [65], psychophysical surround suppression [64, 65], and perceptual learning [68] to assess visual cortex neuroplasticity.

Therefore, in contrast to studies involving animals, studies into a link between exercise and visual cortex neuroplasticity in humans are inconclusive. In the following sections, we identify discrepancies across human studies and suggest how we may reconcile these differences.

## 6. Protocol Discrepancies

Most research into the effects of exercise on vision or visual cortex neuroplasticity in humans incorporates some form of aerobic exercise such as running, jogging, or cycling. However, exercise intensity, exercise dosage, the timing of exercise, and the outcome measure differ across studies.

### 6.1. Exercise Intensity

Many studies incorporate moderate to intense exercise, determined primarily by a target heart rate or a VO2 max measurement. In a recent study of motor cortex plasticity, exercise intensity was found to be an important variable whereby high intensity exercise enhanced neuroplasticity more than moderate exercise [80]. This study measured cortico-motor excitability, short-interval intracortical inhibition, and intracortical facilitation using transcranial magnetic stimulation. Whether this effect of exercise intensity holds for the visual cortex is unknown. For instance, no enhancement of visual cortex neuroplasticity following high intensity exercise has been shown across multiple studies [16, 64, 66, 67], while others found evidence of neuroplasticity enhancement [17, 18, 21, 63]. On the other hand, moderate exercise seems to have no effect on visual cortex neuroplasticity [16, 65, 68]. It is also important to note that each study incorporated the exercise component in a slightly different manner (see Timing: Concurrent vs. Sequential Exercise and Table 1); however, further investigation into whether exercise intensity matters for induction of visual cortex neuroplasticity is warranted.

### 6.2. Exercise Dosage

A key aspect of integrating exercise into a visual perceptual learning protocol is determining how many sessions participants should complete. This is particularly important for potential future clinical applications of the exercise interventions. One session of high intensity exercise, lasting at least 8 minutes, resulted in visual neuroplasticity enhancement seen as a shift in ocular dominance or changes in the concentration of GABA and glutamate [17, 18, 63] (Table 1). A study by Lunghi et al. [79] incorporated six consecutive days of high intensity exercise and reported visual acuity and stereoacuity improvements in adult patients with anisometropic amblyopia that persisted for up to one year when combined with patching. However, as noted above, another treatment was also administered at the same time as the exercise. However, the majority of studies have reported no enhancement in visual cortex plasticity following one session of exercise [16, 64, 65, 67] or multiple sessions of exercise [66, 68]. While the use of consecutive sessions of exercise in humans was adapted from animal model studies that showed longer lasting effects and larger enhancement as compared to a single bout of exercise [46, 81], it remains to be seen whether these results apply to adult humans and are consistent across populations.

### 6.3. Timing: Concurrent vs. Sequential Exercise

While some animal model studies observed enhanced visual cortex plasticity when outcome measures were asynchronous with exercise [46], studies where outcome measures that were synchronous with exercise seem to demonstrate the largest neuroplasticity enhancement. For instance, mice exhibited enhanced detection and discrimination of visual stimuli during exercise but not before or after exercise [39, 44, 81] (Table 1). However, psychophysical measurements during exercise in humans are more difficult to execute. Only one study with humans by Benjamin et al. [65] incorporated concurrent low intensity exercise—specifically walking—while surround suppression was measured psychophysically. Contrary to the animal studies, no enhancement was reported. The logistical complications that arise with the adult human population exercising (running or cycling) at high intensity while focusing on a visual task have logically pushed researchers to take vision measurements either before or after exercise. This may be a reason for the discrepancies between animal and human studies, particularly as exercise either before or after vision measurements or perceptual learning has yielded mixed results; one study even finding that exercise before a vision training session impairs visual perceptual learning [68].

### 6.4. Psychophysical Task

A wide variety of vision tasks and outcome measures have been used by studies of exercise and visual cortex neuroplasticity which may have contributed to the variability in results. Psychophysical tasks that have been employed include binocular rivalry [17, 21, 67], orientation discrimination [63], vernier acuity [66], surround suppression [64, 65], and motion direction discrimination [68]. The variety of psychophysical tasks complicate the comparison of results across studies, particularly since different tasks may recruit differing networks of visual cortical areas (Table 1). It is possible that exercise affects regions of the brain differently depending on their cortical and neurochemical composition. A recent study found that the physiological effects of noninvasive brain stimulation differ across cortical regions [82]. As physical exercise appears to interact with neurochemicals in a similar way to noninvasive brain stimulation, the effects of physical exercise may also differ across various brain regions, influencing distinct visual tasks differently. The relatively recent literature has yet to investigate the impact of exercise on different psychophysical tasks within the same study or assess the visual cortex neurochemical changes before, during, and after physical exercise. In addition, many studies of humans involve young adults who are likely to have a high level of fitness because they have volunteered for an exercise study. Whether baseline fitness level interacts with the effects of exercise on the visual cortex is currently unknown.

## 7. Concluding Remarks and Future Directions

While there is strong evidence from animal models to support the hypothesis that physical exercise can enhance visual cortex plasticity, the results from human studies are variable and are inconclusive. Protocol discrepancies such as exercise intensity, exercise dosage, timing of exercise relative to outcome measures, psychophysical task, and sample population complicate the comparison across the studies. High intensity exercise during perceptual learning may have an increased likelihood of revealing a neuroplasticity enhancement effect as this method shows promising results in animal models. As such, a protocol where visual training can take place while participants exercise intensely should be investigated. If physical exercise indeed influences the human visual cortex and enhances neuroplasticity as seen in animal models, it could be used a rehabilitation treatment for the recovery or improvement of visual function in adults suffering from a wide variety of vision conditions. Further research in this area is warranted.

## Figures and Tables

**Table 1:** Summary table of human studies assessing the effect of exercise on visual function.

Author	Population	Sample size	Visual task	Exercise	Exercise intensity	Measured before/after or during exercise	Exercise dosage	Effect of exercise
Holzschneider et al. [62]	Middle-aged adults (40-55 years)	*n* = 106 (47 included in fMRI)	Spatial learning, virtual maze	45 minutes of cycling	85% max HR	Before/after	Multiple (2x/week for 6 months)	Yes
Lunghi and Sale [17]	Healthy adults (avg age 22)	*n* = 20 (7 males)	Binocular rivalry	10 minutes cycling, 10 minutes off while watching a movie and patching (translucent patch) (2 hours)	~120 bpm	Before/after (up to 120 minutes after patching + exercise)	Single	Yes
Perini et al. [63]	Healthy males (avg age 23)	*n* = 18 exercise group, *n* = 20 control group	Orientation discrimination task	60 revolutions per minute on bike for 30 minutes	70% max HR	Before/after	Single	Yes
Maddock et al. [18]	Healthy adults (avg age~ 25)	*n* = 38	n/a	8-17 minutes of cycling to reach 80% max HR	80% of max HR	Before/after	Single	Yes
Zhou et al. [16]	Healthy adults (avg age 30)	*n* = 10 (3 females)	Binocular combination task	Cycling 10 minutes every 20 minutes for 2 hours	60% or 80% max HR	Before/after (up to 45 minutes after patching + exercise)	Single	No
Benjamin et al. [65]	Healthy adults (avg age 26, 24, 24)	*n* = 13 for behavioral (4 females), *n* = 13 for SSVEP (10 females), *n* = 12 for pupillometry (8 females)	Surround suppression	Walking (5 km/h) vs. standing during visual task	n/a	During	Single	No
Connell et al. [68], Connell et al. [68]	Healthy adults	*n* = 27 (9 per group, M and F)	Motion direction discrimination	Exercise before or after perceptual learning	50% of VO2 max	Before/after	Multiple (5 visits)	No
Lunghi et al. [79]	Adult anisometropic patients (20-40 years)	*n* = 10	Binocular rivalry	Patching during exercise	110-120 bpm for 10 minutes, interleaved for 2 hours	Before/after	Multiple (6 visits)	Yes
Finn et al. [67]	Healthy adults (avg age 22)	*n* = 30 (21 females)	Binocular rivalry	10 minutes cycling, 10 minutes off while watching a movie and patching (translucent patch) (2 hours)	Target HR of 120 bpm	Before/after (up to 120 minutes after patching + exercise)	Single	No
Baldwin et al. [64]	Healthy adults (20-28)	*n* = 20 (3 sessions each)	Dichoptic surround suppression	Cycle for 30 minutes, rest for 90	60% of VO2max	Before/after (up to 45 minutes after intervention)	Single	No
Campana et al. [66]	Healthy adults	*n* = 40, 20 per group (active and control)	Vernier	5 days of moderate exercise	60-70% of max HR based on age	Before/after	Multiple (5 visits)	No
Virathone et al. [21]	Healthy adults (20-43 years)	*n* = 20	Binocular rivalry	10 minutes cycling, 10 minutes off	50-70% of max HR based on age	Before/after (up to 90 minutes after intervention)	Single	Yes
